# Biomarkers of Stress in Companion Animals

**DOI:** 10.3390/ani13040660

**Published:** 2023-02-14

**Authors:** Asahi Ogi, Angelo Gazzano

**Affiliations:** Department of Veterinary Science, University of Pisa, 56124 Pisa, Italy

## 1. Introduction

Stress experienced by companion animals could impair their physical and psychological welfare, impacting their social relationships in domestic environments [1]. For this reason, the establishment of possible reliable biomarkers—objective, quantifiable characteristics of biological processes [2]—is traditionally a goal of scientific research in veterinary behavioral medicine and animal welfare.

The aim of this book was to collect new insight and knowledge on biomarkers as a “characteristic that is measured as an indicator of normal biological processes, pathogenic processes, or biological responses to an exposure or intervention, including therapeutic interventions” [3].

## 2. Cortisol and Dehydroepiandrosterone Sulfate as Possible Biomarkers of Stress during Maternity of Dogs

Cortisol, dehydroepiandrosterone (DHEA) and dehydroepiandrosterone sulfate (DHEA-S) are final products of the activation of the hypothalamic–pituitary–adrenal (HPA) axis. Most previous studies relate to these biomarkers in blood, urine, feces and saliva, but none of these matrices are suitable when long-lasting physiological processes have to be investigated [4]. The use of different matrices, such as claws and hair, has been proposed to overcome these issues, because they provide retrospective information about long-term hormone accumulation [4]. Despite the fact that the oxytocinergic system seems to be triggered by mother–infant interactions [5] and, in turn, oxytocin release could exert inhibitory action on the HPA axis [6], maternity seems to play a crucial role in HPA axis activation. Indeed, maternity in dogs leads to the long-term accumulation of cortisol, but not of DHEA-S, in the coats and nails of female dogs during pregnancy and postpartum [4].

## 3. The Weight of Parturient and Blood Gas Analysis as Possible Biomarkers of Intrauterine Asphyxia in Newborn Canines

Intrapartum hypoxia/asphyxia negatively impacts newborn puppies’ adaptation to extrauterine life [7]. Blood gas analysis allows us to estimate variations in perinatal oxygenation levels, metabolic profiles and the acid–base balance [8]. During eutocic birth, alterations in gases and blood metabolites could indicate respiratory and metabolic acidosis resulting from intrapartum asphyxia [8]. Furthermore, the weight of dams determines the weight of the puppies at birth and, consequently, predicts possible difficulties during expulsion through the birth channel. Indeed, puppies with higher weights have a higher risk of suffering an acute process of intrauterine asphyxia [8].

## 4. Blood Oxytocin, Prolactin, and Serotonin as Possible Biomarkers of Different Lifestyles in Dogs

Oxytocin release in dogs has often been associated with positive experience and, in particular, to positive human–dog interaction [9]. Furthermore, comparing the oxytocinergic system of dogs and wolves, life experience seems to be even more influential on oxytocin levels than domestication [10]. Similarly, the oxytocinergic system of assistance dogs could be more representative of their stressful lifestyle—shaped by repeated separation from their foster family—than their acknowledged prosociality [11]. In fact, even though oxytocin has been frequently linked with more friendliness or sociability, free and total blood oxytocin were found to be surprisingly lower in assistance dogs than pet dogs [11].

Increased levels of prolactin have been previously linked to acute and chronic stress in dogs, but results are conflicting, conceivably because this neurohormone seems to be strongly related to individual variability [11]. In comparison with prolactin, serotonin seems to be a more reliable biomarker. Indeed, low levels of serotonin were found to be associated with stress and behavioral disorders in dogs [12]. Moreover, L-tryptophan (serotonin precursor) [13] and serotonin reuptake inhibitors [14,15] are drugs widely used to treat behavioral problems. However, a clear association of this monoamine with lifestyle in dogs was not found [11], possibly because peripheral serotonin also seems to be affected by dietary patterns [16].

## 5. Facial Expressions as Possible Biomarkers of Emotions in Dogs

Facial expressions, a form of nonverbal communication, not only convey positive and negative emotions, but also have the purpose of contributing to calming/de-escalating other interacting dogs [17]. Despite the fact that the literature regarding possible tools for standardizing the Facial Action Coding System (FACS) is quite extensive, the association with underlying emotions is still difficult to evaluate on an objective basis [18]. The interspecies and the interbreed variability of facial mimicry, in fact, makes the standardization very complex [18]. However, through the FACS, some veterinary tools (e.g., the Grimace Scale) are currently available to help clinicians recognize pain and distress in various domestic animals [18]. These tools are crucial in improving the interpretation of animal body language and therefore the attitude of veterinarians towards animal welfare [19].

## 6. Skin Temperature as Possible Biomarker of Stress in Companion Animals

Stressful events are known to trigger sympathetic activation which, in turn, could cause an increase in body temperature. Infrared thermography (IRT) exploits a specific camera to detect the radiation spectrum and visualize changes in skin temperature which can be correlated with possible health/stress conditions in companion animals [20]. IRT is a safe and non-stressful technique, but currently, it is difficult to say that the evidence of its reliability is conclusive, mostly because of the possible influence of environmental factors [20].

## 7. Conclusions

Oxytocin, cortisol, DHEA-S, prolactin, serotonin, facial expressions, skin temperature, and birthweight are just some of possible biomarkers of stress in companion animals. In addition, all of these parameters could also be influenced by many other variables, such as the traits of the subjects (age, sex, breed, temperament, etc.) and methodological approaches (matrix, assay, instrumentation, etc.). In light of this complex picture, the present topic needs and deserves further investigations to optimally manage the stress experienced by companion animals and improve their welfare.
